# Desmoplastic Small Round Cell Tumor: A Rare Location in the Parotid Gland

**DOI:** 10.7759/cureus.10068

**Published:** 2020-08-27

**Authors:** Elisabeth Ninchritz-Becerra, Jose González-García, Leire García-Iza, Carlos M Chiesa Estomba

**Affiliations:** 1 ENT - Head & Neck Surgery, Hospital Universitario Donostia, Donostia, ESP

**Keywords:** desmoplastic small round cell tumor, parotid gland, gracilis muscle free flap

## Abstract

Desmoplastic small round cell tumor represents an unusual variety of tumors that affects mostly children and young males. In this report we present a case of a desmoplastic small round cell tumor involving the parotid gland of a young male. The tumor was excised, and definitive histology showed features of desmoplastic small round cell tumor confirming the diagnosis.

## Introduction

Desmoplastic small round cell tumor (DSRCT) represents an unusual variety of tumors that affects mostly children and young males. The peak age of incidence is around the third decade of life and primary locations are usually soft tissues of the abdomen or pelvis, like retroperitoneum, omentum or mesenteries [1]. DSRCT cases in major salivary glands have been reported but in a very small number of patients. The origin of this tumor is not well-known, and some authors hypothesize that it could derive from mesothelial or submesothelial cells because of the usual location and because of the positivity for antigens like desmin and Wilms tumor 1 (WT-1) [2]. In this report, we present a case of a DSRCT involving the parotid gland of a young male.

## Case presentation

An 18-year-old man without medical background noticed a non-painful mass in the left parotid gland, with no other symptoms, for less than a month. No history of weight loss, fever or night sweats was reported. In the physical examination, a mobile, non-tender oval mass with a 1.5 cm diameter approximately on the left parotid gland, was found. There was no facial nerve dysfunction.

**Figure 1:**
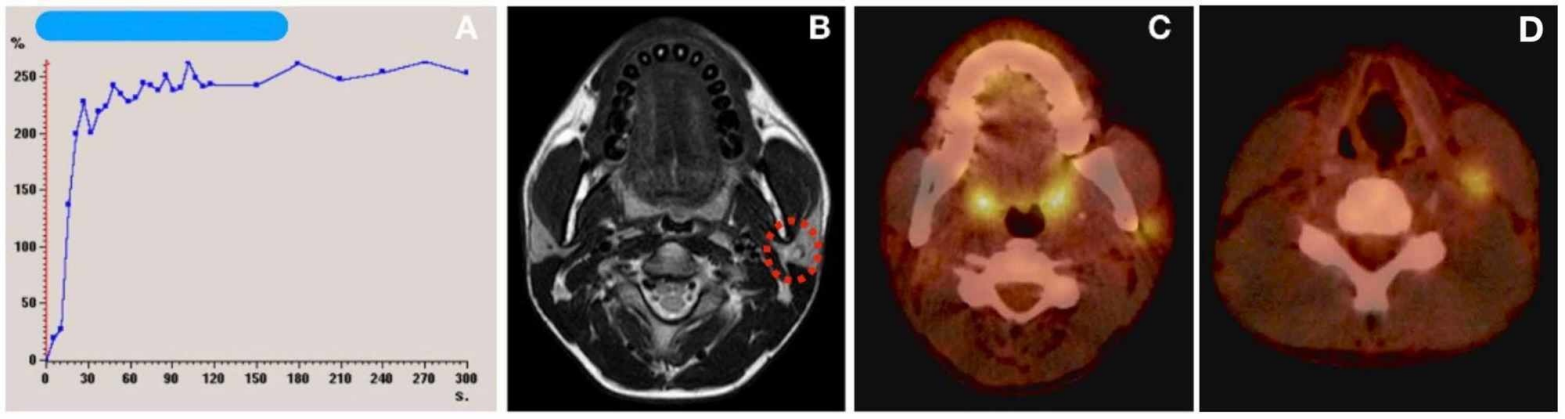
A) DWI demonstrate a type III ADC curve with an early rise and moderate contrast wash suggesting of malignancy; B) T2 weighted image MRI with a lesion in the left parotid mass - red dotted circle (1.5-T system; Ingenia, Philips Healthcare Systems, The Netherlands); C) PET-CT scan with a left parotid mass; D) PET-CT scan with a lymph node in the level II DWI: diffusion weight image; ADC: apparent diffusion coefficient; PET-CT: positron emission tomography CT

Ultrasound (US) examination demonstrated a solid, polylobed and poorly-defined contour mass, about 21 mm in diameter, with small hyperechogenic focus inside, located in left parotid gland. T1-weighted magnetic resonance imaging showed a low-density and poorly-defined mass, involving both parotid lobes without extra-parotid extension, with high-intensity in T2-weighted sequences and low apparent diffusion coefficient (ADC) score (0.8) (Figure 1). Diameter estimated was 12 x 15 x 30 mm. Accompanied by one cervical lymph node suspicious of malignancy (Figure 1). A positron emission tomography scan CT (PET-CT) confirmed a hypermetabolic left parotid focus and a left latero-cervical node (Figure 1). Fine-needle aspiration biopsy was performed of the parotid mass and the lymph node. Cytologically, the aspirate from both were very cellular with small, round monotonous cells with hyperchromatic nuclei, little nucleoli, and scanty cytoplasm. Occasionally, small clusters of cells with poor cohesiveness were observed in which cytoplasms that appear weakly basophilic were better identified. Apoptotic cells and high mitotic activity were observed. Small collagenized stromal fragments were seen very occasionally. Periodic acid-Schiff stain (PAS) stain was negative. Immunohistochemistry was performed on Papanicolaou-stained smears (PSS). Tumor cells were positive for monoclonal mouse anti-human cytokeratin, clones AE1/AE3 (CK AE1/AE3) and desmin, the latter showed cytoplasmic and perinuclear dot-like positivity. Cluster of differentiation 99 (CD99), myogenic differentiation 1 (MYO-D1), and myogenin were negative. Due to the high suspicion of desmoplastic small round cell tumor, a fluorescence in situ hybridization (FISH) technique was performed on PSS detecting the reciprocal chromosomal translocation t(11;22) (p13;q12). The diagnosis of DSRCT was then confirmed.

**Figure 2:**
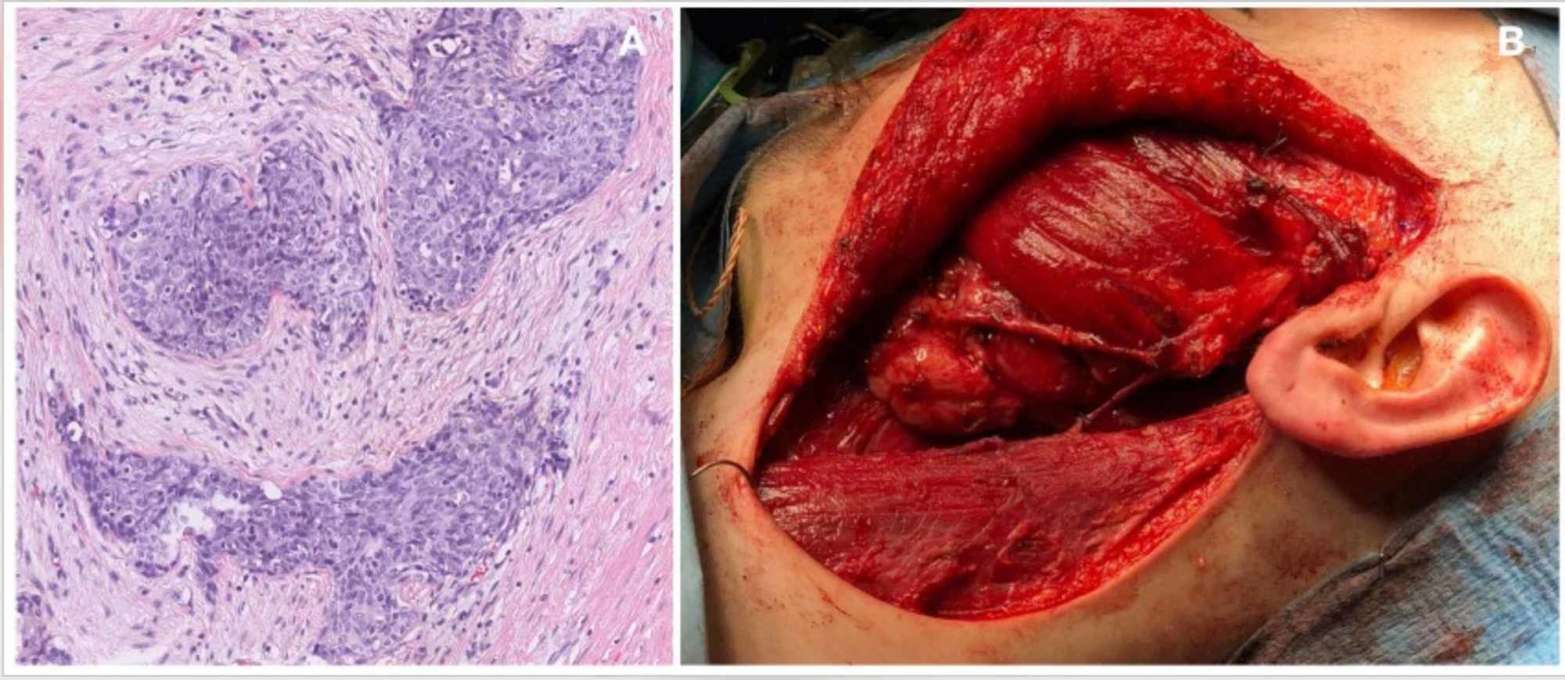
A) Histological image of a desmoplastic small round cell tumor: infiltrating the parotid gland parenchyma, composed by tumoral nests of blue cells within a fibrotic stroma with scant cytoplasm and medium-sized cells with clear cytoplasm; B) Surgical result after radical parotidectomy, left neck dissection and gracilis free flap reconstruction

The patients received vincristine sulfate, adriamycin and cyclophosphamide, followed by ifosfamide and etoposide phosphate (VAC-IE) as neoadjuvant chemotherapy, following which radical surgery and adjuvant chemoradiotherapy were recommended after discussions in the head and neck cancer committee. A left radical parotidectomy, due to gross facial nerve involvement evidenced during surgery with cervical lymph nodes dissection of level II and III, was performed. Facial nerve reconstruction was performed at the same sitting, and a free gracilis muscle flap was transferred by splitting the obturator nerve to perform two coaptation to both free ends of the facial nerve (through the sural graft) and directly to the masseteric nerve (Figure 2). Finally, a left palpebral gold weight was implanted. The definitive histological analysis showed a tumor with well-defined border and some focal infiltrative areas and multiple cellular nests growing in a paucicellular fibromyxoid stroma. Tumor cells were round to polygonal and small- to medium-sized; cytoplasm was irregular and accompanied with granular chromatin. Rhabdoid cells or necrosis were not evident. There was presence of some non-neoplastic salivary ducts between tumor nests, demonstrating that the tumor infiltration within the parotid parenchyma. One metastatic lymph node of 1 cm, without extra capsular extension was found (Figure 2). 

A good initial response to treatment was observed, including a good functional outcome of the facial nerve reconstruction (House-Brackman 3/6). The patient received adjuvant chemoradiotherapy with a good response. After 14 months of follow-up, a retropharyngeal lymph node recurrence was observed and confirmed by histology. Actually, the patient has underwent a divergent cycle of chemotherapy.

## Discussion

Desmoplastic small round cell tumor represents an unusual variety of tumors that affect mostly children and young males. The peak age of incidence is around the third decade of life and primary locations are usually soft tissues of the abdomen or pelvis, like retroperitoneum, omentum or mesenteries [1]. Extra-abdominal presentations are infrequent [1]. 

DSRCT cases in major salivary glands have been reported but in a very small number of patients. The most common mesenchymal salivary gland tumor described across the literature is the lipoma, but malignant histology is uncommon [3]. About differential diagnosis, this type of tumor is not usually included as a primary salivary gland tumor. Other diagnoses like malignant melanoma, Merkel cell carcinoma, metastatic neuroblastoma, Ewing sarcoma, rhabdomyosarcoma or malignant lymphoma are more common. Also, we should differentiate it from a solid variant of an adenoid cystic carcinoma [2].

Primary small cell undifferentiated carcinoma of the salivary glands represents around 2% of all salivary gland tumors and are more common in older patients than the DSRCT. Molecular biology studies are very useful in the differentiation between these tumors [2,4]. Primary sarcomas of the major salivary glands are very uncommon, around 0.3-0.5%, including all the salivary gland tumors, and 1.5-2.3% of malignant salivary tumors [3].

The histology of this neoplasm is very characteristic, usually composed of nests of small round blue cells with a prominent desmoplastic, fibromyxoid or collagenous stroma [2]. The immunohistochemical profile is essential for diagnosis and in general DSRCT shows a striking pattern of multi-phenotypic differentiation; a dot-like staining pattern of desmin and positive nuclear staining with WT1 antibody are typical. The detection of the EWSR1-WT1 gene fusion, a specific translocation t(11;22) (p13;q12), is very useful in the differential diagnosis and confirmation of this tumor. Also, this allow pathologies to differentiate the DSRCT from other small cell blue tumors like the Ewing sarcoma. In case of an atypical DSRCT presentation, due to lack of WT1 immunoreactivity, we should consider other aspects like clinical, morphologic and immunohistochemical features [2,3,5,6].

However, DSRCT has a poor overall survival despite initially possible good response to a multimodal treatment protocol, including surgery, chemotherapy and radiotherapy [2]. To the best of our knowledge, the elective treatment for head and neck sarcomas has not been standardized yet [3]. Satisfactory responses to the treatment could not persist more than three years after the diagnosis [5].

## Conclusions

Although DSRCT in the major salivary glands is extremely rare, it should be included in the differential diagnosis of poorly differentiated salivary gland tumors, especially in adolescent and young adult males. Survival is low despite their initial response to multimodal treatment. Most patients relapse with disseminated disease that is unresponsive to further therapy.
